# Ror2 modulates the canonical Wnt signaling in lung epithelial cells through cooperation with Fzd2

**DOI:** 10.1186/1471-2199-9-11

**Published:** 2008-01-23

**Authors:** Changgong Li, Hongyan Chen, Lingyan Hu, Yiming Xing, Tomoyo Sasaki, Maria F Villosis, John Li, Michiru Nishita, Yasuhiro Minami, Parviz Minoo

**Affiliations:** 1 Department of Pediatrics, Division of Neonatology, University of Southern California Keck School of Medicine, Los Angeles, CA 90033, USA; 2 Department of Physiology and Cell Biology, Faculty of Medical Sciences, Graduate School of Medicine, Kobe University, Kobe 650-0017, Japan

## Abstract

**Background:**

Wnt signaling is mediated through 1) the beta-catenin dependent canonical pathway and, 2) the beta-catenin independent pathways. Multiple receptors, including Fzds, Lrps, Ror2 and Ryk, are involved in Wnt signaling. Ror2 is a single-span transmembrane receptor-tyrosine kinase (RTK). The functions of Ror2 in mediating the non-canonical Wnt signaling have been well established. The role of Ror2 in canonical Wnt signaling is not fully understood.

**Results:**

Here we report that Ror2 also positively modulates Wnt3a-activated canonical signaling in a lung carcinoma, H441 cell line. This activity of Ror2 is dependent on cooperative interactions with Fzd2 but not Fzd7. In addition, Ror2-mediated enhancement of canonical signaling requires the extracellular CRD, but not the intracellular PRD domain of Ror2. We further provide evidence that the positive effect of Ror2 on canonical Wnt signaling is inhibited by Dkk1 and Krm1 suggesting that Ror2 enhances an Lrp-dependent STF response.

**Conclusion:**

The current study demonstrates the function of Ror2 in modulating canonical Wnt signaling. These findings support a functional scheme whereby regulation of Wnt signaling is achieved by cooperative functions of multiple mediators.

## Background

WNT ligands are a family of secreted cysteine-rich signaling molecules that play critical roles in many cell activities, including cell fate determination, cell adhesion, cell migration and cell polarity. Several pathways have been identified that mediate Wnt signaling. The most extensively studied of these, the beta-catenin-dependent, otherwise known as the canonical Wnt pathway is activated through stabilization of beta-catenin, followed by beta-catenin nuclear-localization and activation of target genes by beta-catenin-LEF/TCF complex [1,2]. Wnt ligands also stimulate beta-catenin-independent, or the non-canonical Wnt pathways that include the planer cell polarity (PCP), and the Wnt/Ca2+ pathways. The PCP pathway is mediated by activation of RhoA and JNK, and the Wnt/Ca2+ pathway leads to activation of protein kinase C (PKC), calcium-calmodulin dependent kinase II (CamKII) or calcineurin (CaCN) [2,3].

To activate the intracellular pathways, Wnt ligands interact with seven-span transmembrane receptor molecules known as Frizzled (Fzd), and the co-receptors from the family of low-density lipoprotein receptor-related proteins (Lrp). Fzd proteins contain a cysteine-rich domain (CRD) in their N-terminal extracellullar region, which interacts with Wnt ligands. To date, 19 WNT ligands, 10 Fzds and 2 Lrps have been described in human tissues. Different combinations of Wnt and Fzds lead to differential responses. For example, Wnt5a can activate or inhibit the canonical Wnt signaling depending on the availability of specific receptors [4]. Recently, studies on the mechanism of Wnt signaling suggested that coupling of Fzd and Lrp triggers the activation of the canonical Wnt pathway [5].

Ror2, a member of the Ror family of receptor-tyrosine kinases (RTKs), was first identified as a receptor tyrosine kinase-like orphan receptor. Ror2 is a single-span transmembrane receptor that contains a CRD domain in the extracellular region. The C-terminal intracellular region of Ror2 contains a tyrosine kinase domain (TK) and a proline-rich domain (PRD) flanked by two Ser/Thr rich domains (S/T1 and S/T2) [6]. The PRD of Ror2 associates with casein kinase 1ε (CK1ε) which catalyzes phosphorylation of Ror2 at S/T2. This in turn leads to Ror2 auto-phosphorylation at tyrosine residues in PRD [6]. Targeted deletion of Ror2 gene resulted in abnormal skeletal, genital and cardiovascular development [7,8]. The potential role of Ror2 in Wnt signaling has been the focus of many recent studies. For example, the Ror2 CRD domain has been shown to interact with Wnt ligands and the CRD domain of Fzd2 [9]. Minami and his colleagues first demonstrated that Ror2 mediates Wnt5a-induced activation of the non-canonical pathway [9]. Subsequently, Ror2 was found to mediate the inhibitory effect of Wnt5a on Wnt3a-mediated activation of the canonical Wnt pathway [4]. More recently, Ror2 was shown to be critical for filopodia formation during cell migration, a process dependent on Wnt5a. Knowledge on the role of Ror2 in canonical Wnt signaling is limited. To date, one study has reported potentiation of Wnt1 by Ror2 in activating the canonical Wnt pathway in osteoblastic cells [10].

Wnt signaling plays important roles in lung development and lung cancer [11-18]. We have shown that Wnt5a is a key regulator of epithelial-mesenchymal interaction during lung development. Lungs from Wnt5a (-/-) and transgenic embryos that over-express Wnt5a specifically in lung epithelial cells are abnormal in branching morphogenesis and lung maturation [11,17]. Targeted inactivation of Wnt7b disrupts mesenchymal proliferation and vascular development in the lung [12]. Studies on Wnt inhibitor, Dickkopf-1 (DKK1), suggested that Wnt signaling controls fibronectin deposition during branching morphogenesis of the lung [19].

To further understand the mechanism of Wnt signaling in the lung, we analyzed it in a lung carcinoma H441 cell line, which as we found in this report, has low levels of Fzd expression. We show that in H441 cells, Ror2 moderately enhances activation of canonical Wnt pathway by Wnt3a. Also, Ror2 specifically cooperates with Fzd2 but not Fzd7 in mediating Wnt3a-stimulation of the canonical Wnt pathway. The cooperative function between Ror2 and Fzd2 is dependent on the CRD, but independent of the PRD domain of Ror2. Furthermore, we demonstrate that Lrp5/6 is also critical for the cooperative function of Ror2 and Fzd2, suggesting that Ror2 functions as a cofactor to facilitate Fzd2 and Lrp5/6-mediated activation of the canonical Wnt signaling pathway.

## Results

### Activation of the canonical pathway by Wnt3a in lung epithelial cell lines

**Figure 1:**
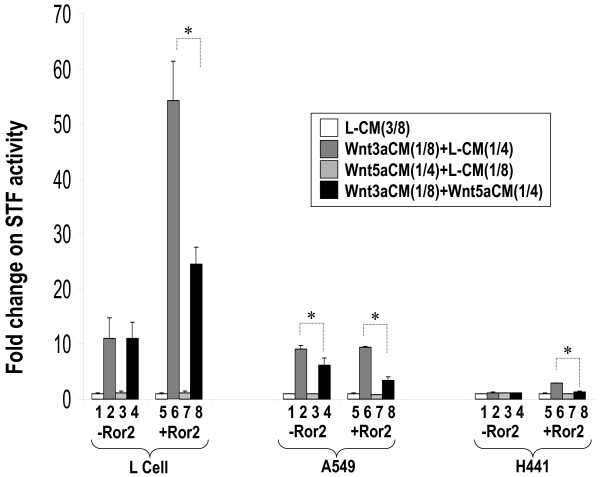
**Effects of Wnt3aCM and Wnt5aCM on Canonical Wnt signaling in L, A549, and H441 cells**. Cultured L, A549, or H441 cells were transfected with *pSV-β*-*gal *(Promega, WI) and STF plus Ror2WT (+Ror2) or the control vector (-Ror2). Twenty four hours after transfection, cells were treated with L-CM, Wnt3aCM or Wnt5aCM. Wnt3aCM and Wnt5aCM were used at 1/8 and 1/4 dilutions, respectively. Equal amount of conditioned media was used in each assay. Luciferase values representing the STF activities were normalized to beta-galactosidase for transfection efficiency. Values of the assays treated with L-CM alone (#1 and #5) were adjusted to unity to normalize all other experimental values in the same group. CM: conditioned medium. * indicates p < 0.05.

Wnt signaling plays important roles during lung development [11-18] and in non-small-cell lung cancer [15]. To understand its mechanism, we examined Wnt signaling in two lung carcinoma, A549 and H441 cell lines. Both cell lines were transiently transfected with the canonical Wnt signaling reporter SuperTopFlash, STF, that contains the luciferase coding region driven by a minimal TA viral promoter, plus eight LEF/TCF response elements. These cells were then treated with either Wnt3a- or Wnt5a-conditioned media (ATCC, Manassas, VA). These conditioned media have been previously used as a source of recombinant Wnt ligands [20,21]. As positive control, an identical experiment was conducted in L cells, in which both Wnt3a and Wnt5a have been shown to function in regulating STF activity [4]. As shown in Figure 1, Wnt3a stimulated STF activity in both L cells and in A549 cells. In contrast, only a low level of STF response to Wnt3a was observed in H441 cells. We also used an experimental approach previously used by Mikels and Nusse [4] to determine the activity of Wnt5a by examining its ability to antagonize Wnt3a-mediated STF response. This antagonistic effect was shown to be enhanced by the presence of Ror2 [4]. In all three cell lines, stimulation of STF by Wnt3a was antagonized by Wnt5a in presence of Ror2 (Figure 1). Unexpectedly, in both L cells and H441 cells, presence of Ror2 enhanced Wnt3a-stimulated STF activity. Thus, the results of these experiments show that: 1) the canonical Wnt signaling pathway as assessed by the magnitude of response to Wnt3a-stimulated STF activation is significantly reduced in H441 cells compared to L cells or A549 cells and 2) Ror2 may positively modulate the canonical Wnt signaling in L and H441 cells.

### Low Level Frizzled Expression in H441 Cells

**Figure 2:**
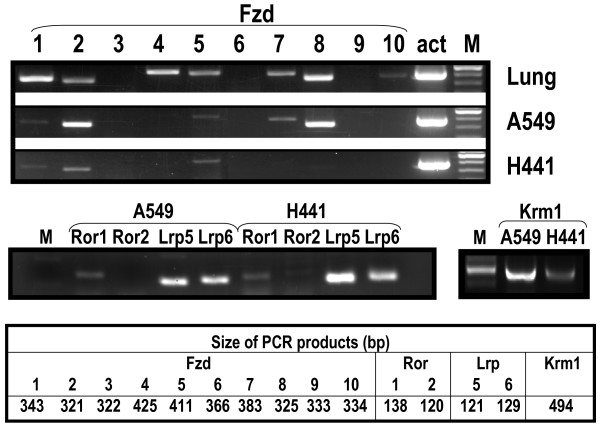
**Expression of Wnt signaling receptors in the adult lung and lung carcinoma cell lines**. Expression of Fzds in A549 and H441 cells was determined by RT-PCR. For comparison, expression in human lung tissue was also determined. Expected size of each PCR product was listed in the table. Both Lrp5 and Lrp6 are expressed in A549 and H441 cells. Of the two Ror family members, only Ror1 is detectable.

One potential mechanism by which the "low responsiveness" of H441 cells to Wnt3a or Wnt5a may be explained is the lack of appropriate receptors. Therefore, we examined the expression of all 10 Fzd molecules and some of the Wnt related membrane-associated cofactors in A549 and H441 cells by RT-PCR analysis. For comparison, we also examined the expression of these molecules in human lung tissue. Since many Fzd genes, (e.g. Fzd1, Fzd2) do not contain intronic sequences, the products from the reverse-transcription reactions without adding reverse-transcriptase (NO-RT) were used as negative controls in each PCR amplification to eliminate the possibility of genomic DNA contamination. As shown in Figure 2, most of the Fzds, except Fzd3, 6, and 9 are expressed in human lung. Fzd1, 2, 5, 7 and 8 are detectable in A549 cells. Fzds not detected in human lung were also not expressed in A549 cells. Expression of Fzds in L cells was determined previously [22]. Fzds 1, 2, 4, 5, and 7 are expressed in L cells. Interestingly, in H441 cells, expression of Fzds was nearly undetectable. Only weak bands were observed corresponding to Fzds1, 2, and 5. No product was amplified when samples without RT were used in PCR reactions (data not shown). Expression patterns of other Wnt related transmembrane molecules such as Lrp5, Lrp6, Ror1 and Ror2 were similar between A549 and H441 cells, while level of Krm1 in H441 appeared much lower than in A549 cells. Therefore, H441 cells express not only fewer types of Fzds but also at reduced levels, which may explain the absence or reduced response of these cells to Wnt3a ligand. These characteristics distinguish H441 as a useful lung epithelial cell model to study Wnt ligand-receptor interactions and signaling.

### Transient Fzd2 and Fzd7 Augmentation Enhances H441 Responsiveness to Wnt3a

**Figure 3:**
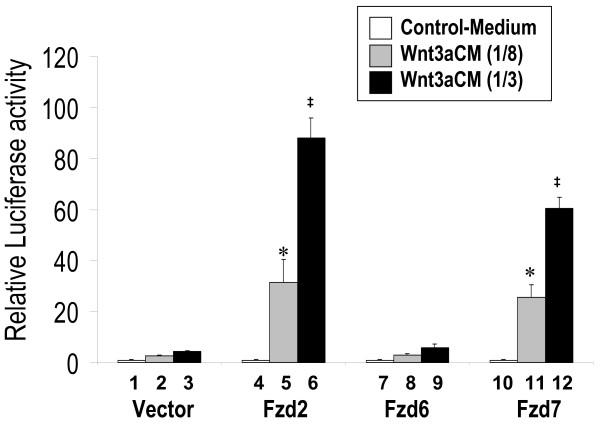
**Fzd2 and Fzd7, but not Fzd6 mediate Wnt3a stimulation of canonical Wnt signaling**. H441 cells were transfected with *pSV-β*-*gal *(Promega, WI) and STF, plus expression constructs for Fzd2, Fzd6, or Fzd7. An equal quantity of control vector was used as negative control. Twenty-four hours after transfection, cells were treated with L-CM and Wnt3aCM. Two dilutions of Wnt3aCM (1/8 or 1/3) were applied. Equal amounts of conditioned media were used in each assay. Luciferase values representing STF activities were normalized to beta-galactosidase for transfection efficiency. Values of the assays treated with L-CM alone were adjusted to unity to normalize all other experimental values in the same group. * indicates p < 0.05 when compared with data of bar 2. **‡ **indicates p < 0.05 when compared with data of bar 3.

**Figure 4:**
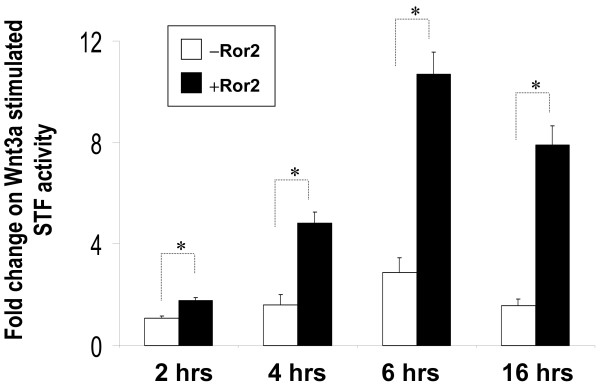
**Ror2 potentiates Wnt3a stimulation of canonical Wnt signaling**. H441 cells were transfected with *pSV-β*-*gal *(Promega, WI) and STF, plus expression constructs for Ror2 wild-type (Ror2WT). An equal quantity of control vector was used as negative control. Twenty-four hours after transfection, cells were treated with L-CM and Wnt3aCM for 2 hrs, 4 hrs, 6 hrs or 16 hrs. Luciferase values representing STF activities were normalized to beta-galactosidase for transfection efficiency. Data shown are the average ratios of normalized luciferase values in Wnt3aCM-treated cells over that of L-CM-treated cells. * indicates p < 0.05.

The latter observations suggested that low level expression of endogenous Fzd receptors might be responsible for the low-responsiveness of H441 cells to Wnt3a and Wnt5a signaling. To validate this possibility, we transiently expressed Fzd 2 and Fzd7, two receptors shown to mediate Wnt3a signaling in 293 cells [5], along with STF in H441 cells. Transfected cells were then treated with Wnt3a- or Wnt5a-conditioned media. In comparison to controls, transfected H441 cells responded strongly to Wnt3a stimulation as demonstrated by the magnitude of STF activation (Figure 3). In contrast, transient transfection of a Fzd6 expression construct failed to establish or enhance Wnt3a responsiveness in H441 cells. None of the transfected Fzds activated STF by Wnt5a (data not shown). Ror2 is known to mediate the non-canonical Wnt signaling and the antagonistic effect of Wnt5a on canonical Wnt signaling. However, in Figure 1 we found that Ror2 enhanced the Wnt3a-stimulated STF activation in both L and H441 cell lines. To confirm this observation, we treated H441 cells transfected with Ror2 expression constructs with Wnt3a-conditioned medium for different time periods. As shown in Figure 4, Ror2 moderately potentiated stimulation of STF when H441 cells were treated with Wnt3a for durations from 4 hrs to 16 hrs. Ror2 slightly enhanced STF stimulation when H441 cells were treated for 2 hours.

### Fzd2 and Ror2 Cooperate to Mediate Wnt3a-activation of the Canonical Wnt Pathway

**Figure 5:**
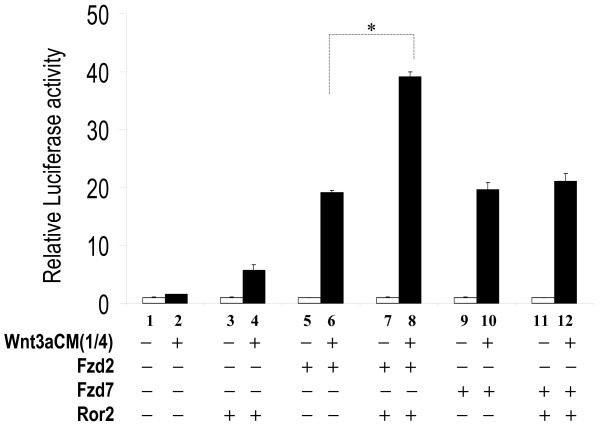
**Ror2 cooperates with Fzd2 but not Fzd7 in mediating Wnt3a-stimulated canonical Wnt signaling in H441 cells**. H441 cells were transfected with *pSV-β*-*gal *(Promega, WI), STF, plus either Fzd2 or Fzd7 expression constructs with or without Ror2WT plasmid and then treated with conditioned media, L-CM or Wnt3aCM. Equal amounts of DNA and conditioned media were used in each assay. Luciferase values were normalized to beta-galactosidase values for transfection efficiency. Values of the assays treated with L-CM alone (empty bars) were adjusted to unity to normalize all other experimental values in the same group. * indicates p < 0.05.

**Figure 6:**
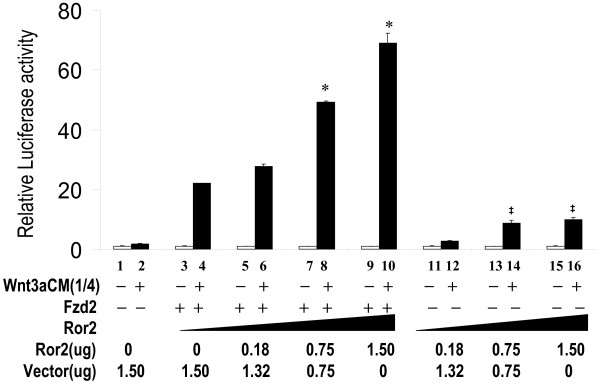
**Response of STF to increasing dose of Ror2 in H441 cells**. H441 cells were transfected with *pSV-β*-*gal *(Promega, WI), STF, Fzd2 expression construct and increasing doses of Ror2 wild-type (Ror2WT) plasmid, and then treated with conditioned media, L-CM or Wnt3aCM. Equal amounts of DNA and conditioned media were used in each assay. Values of the assays were normalized as in Fig. 5. * indicates p < 0.05 when compared with data of bar 4. **‡ **indicates p < 0.05 when compared with data of bar 2.

To further determine the role of Ror2 in the canonical Wnt pathway, we transfected H441 cells with Ror2, plus Fzd2 or Fzd7 expression constructs and treated the transfected cells with Wnt3a conditioned medium. As shown in Figure 5, Ror2 alone lead to nearly a 5 fold increase in Wnt3a-stimulated STF response, while Fzd2 alone stimulated a 20 fold increase. Interestingly, combination of Fzd2 and Ror2 resulted in a significant further increase in STF response. In contrast, although Fzd7 alone mediated similar level of Wnt3a-stimulated STF response as Fzd2, co-transfected Ror2 with Fzd7 failed to further increase the level of response (Figure 5). Similar levels of Ror2 expression were observed by western blot analysis in cells transfected with Ror2 alone, Ror2 plus Fzd2, or Ror2 plus Fzd7 (data not shown). The affinity of Ror2 to Fzd2 CRD domain is significantly higher than that of Fzd7 CRD domain [see Additional file 1]. To further determine the cooperative effects between Fzd2 and Ror2, we performed the transfection assay with increasing doses of Ror2. As shown in Figure 6, the levels of STF response to Wnt3a increased in response to increased doses of Ror2, suggesting cooperation between Ror2 and Fzd2 inWnt3a-stimulated STF activity. Also shown in Figure 6, Ror2 alone can also lead to moderate activation of STF by Wnt3a. These observations suggest that 1) Ror2 positively modulates activation of the canonical Wnt pathway and 2) Ror2 cooperatively enhances Fzd2 but not Fzd7 in this reaction. The latter functions of Ror2 and Fzd2 were also observed when purified recombinant Wnt3a protein (R&D systems) was used (Data not shown).

### Cooperative Function of Ror2 and Fzd2 is Dependent on Ror2 CRD Domain, but Independent of Ror2 PRD Domain

**Figure 7:**
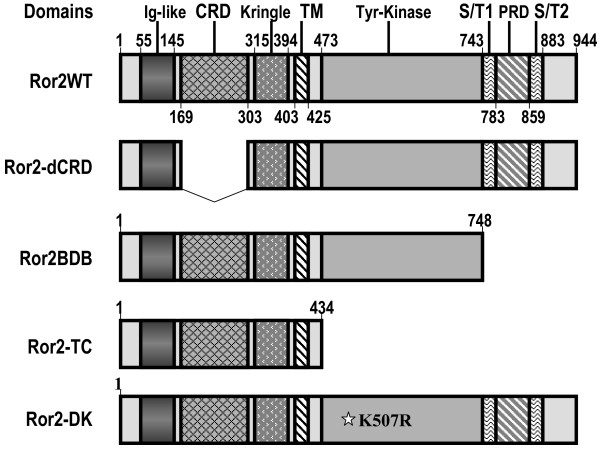
**Schematic representation of the wild-type (Ror2WT) and mutant derivatives of Ror2**. Numbers indicate amino acid positions. Major functional domains of Ror2 are indicated. CRD: cysteine-rich domain; TM: transmembrane domain; Tyr-Kinase: tyrosine-kinase domain; S/T: serine and thronine rich domain; PRD: proline-rich domain.

**Figure 8:**
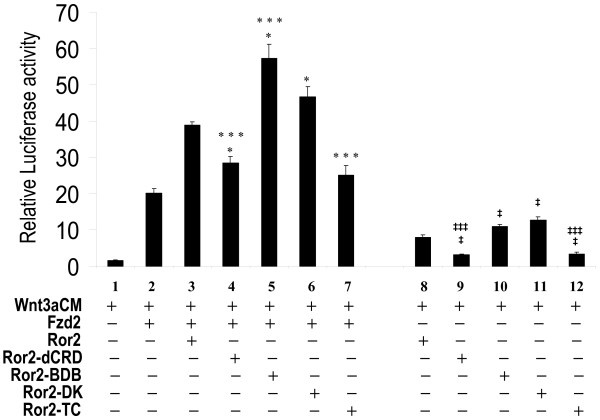
**The CRD and not the PRD domain of Ror2 is required for Ror2-Fzd2 cooperation**. H441 cells were transfected with *pSV-β*-*gal *(Promega, WI), STF, Fzd2 and expression constructs for Ror2 wild-type and mutant derivatives, and then treated with L-CM or Wnt3aCM. Equal amounts of DNA and conditioned media were used in each assay. Luciferase values were normalized to beta-galactosidase values for transfection efficiency. Data shown are the average ratios of normalized luciferase values in Wnt3aCM-treated cells over that of L-CM-treated cells. * indicates p < 0.05 when compared with data of bar 2. *** indicates p < 0.05 when compared with data of bar 3. **‡ **indicates p < 0.05 when compared with data of bar 1. **‡‡‡ **indicates p < 0.05 when compared with data of bar 8.

**Figure 9:**
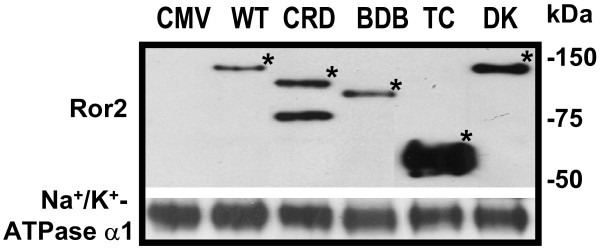
**Relative levels of Ror2 mutant peptides in H441 cell membrane determined by western blot**. Positions of the respective Ror2 mutant proteins were indicated by asterisks. Two distinct bands were detected for Ror2-dCRD, presumably reflecting alternative modification (e.g. glycosylation).

To determine which of the Ror2 domains is involved in the cooperative function of Ror2 and Fzd2, we used several Ror2 mutants that lack specific functional domains. Oishi et al [9] has demonstrated that Fzd2 and Ror2 physically interact with each other through their CRD domains. Ror2-dCRD lacks the CRD domain and hence is unable to interact with Fzd2 CRD [9]. To determine whether the cooperative function of Ror2 is dependent on its CRD domain, we conducted the latter experiments with Ror2-dCRD. As expected, Ror2-dCRD failed to fully cooperate with Fzd2 in mediating Wnt3a-stimulated STF response, suggesting that interaction with the CRD domain is important for the cooperative function between Ror2 and Fzd2. However, deletion of the CRD domain did not completely abolish the cooperative effect of Ror2. Ror2 also contains a Proline-rich domain, PRD, localized to the intracellular region of the molecule. PRD is important in phosphorylation of G protein-coupled receptor kinase 2 [6] and Ror2-mediated cell migration [23]. Interestingly, Ror2-BDB (Figure 7), an Ror2 mutant that lacks the PRD domain and the Ser/Thr rich domains flanking PRD, functioned even more efficiently than the wild-type Ror2, suggesting that the Ser/Thr rich domains and the PRD domain are not required for cooperating with Fzd2. Further experiments using the mutant Ror2-DK construct that lacks the Tyr-kinase activity by amino acid substitution [23] showed that the tyrosine kinase activity of the Ror2 intracellular region does not affect Fzd2-Ror2 cooperativity (Figure 8). However, Fzd2-Ror2 cooperation was entirely abolished by using Ror2-Tc, a mutant which lacks the entire intracellular domain of Ror2. Relative level of Ror2 mutant peptides in H441 cell membrane was determined by western blot (Figure 9).

### Fzd2-Ror2 Mediated Activation of STF by Wnt3a is Inhibited by Dkk1-Krm1 and GSK3

**Figure 10:**
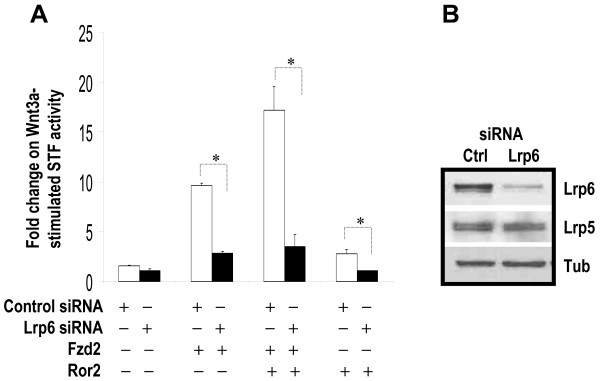
**Wnt3a stimulation of STF mediated by Fzd2 and Ror2 is inhibited by Lrp6 siRNA**. **A**. H441 cells were transfected with *pSV-β*-*gal *(Promega, WI), STF, Fzd2 and Ror2WT with either Lrp6 siRNAs or negative control siRNAs, and then treated with conditioned media, L-CM or Wnt3aCM. Equal amounts of DNA, siRNAs and conditioned media were used in each assay. Luciferase values were normalized to beta-galactosidase for transfection efficiency. Data shown are the average ratios of normalized luciferase values in Wnt3aCM-treated cells over that of L-CM-treated cells. * indicates p < 0.05. **B**. Western blot analysis of levels of Lrp6, Lrp5 and alpha-tubulin in H441 cells transfected with either Lrp6 siRNA or negative control siRNA (Ctrl).

**Figure 11:**
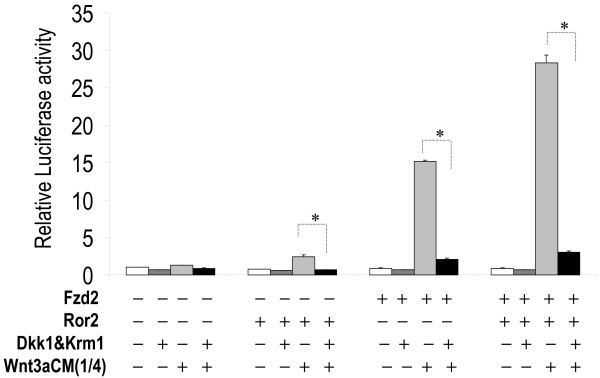
**Wnt3a stimulation of STF mediated by Fzd2 and Ror2 is inhibited by Dkk1 and Krm1**. H441 cells were transfected with *pSV-β*-*gal *(Promega, WI), STF, Fzd2 and Ror2WT with and without Dkk1 and Krm1 expression constructs, and then treated with conditioned media, L-CM or Wnt3aCM. Equal amounts of DNA and conditioned media were used in each assay. Luciferase values were normalized to beta-galactosidase for transfection efficiency. Values of the assay transfected with control vector and treated with L-CM alone (empty bars) were adjusted to unity to normalize all other experimental values in the same group. * indicates p < 0.05.

**Figure 12:**
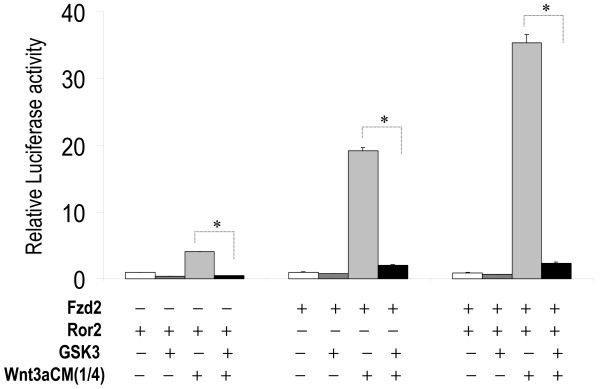
**Wnt3a stimulation of STF mediated by Fzd2 and Ror2 is inhibited by GSK3**. H441 cells were transfected with *pSV-β*-*gal *(Promega, WI), STF, Fzd2 and Ror2WT plus Xgsk3 expression construct, and then treated with L-CM or Wnt3aCM. Equal amounts of DNA and conditioned media were used in each assay. Luciferase values were normalized to beta-galactosidase for transfection efficiency. Values of the assay transfected with control vector and treated with L-CM alone (empty bars) were adjusted to unity to normalize all other experimental values in the same group. * indicates p < 0.05.

To further determine if Ror2 potentiates Wnt3a-activation of STF through activation of canonical Wnt signaling, we examined whether Lrps are required. We first performed transfection assays with siRNAs that specifically target Lrp6. Specificity of the Lrp6 siRNAs was determined by western blot analysis (Figure 10B). As shown in Figure 10A Lrp6 siRNAs almost completely abolished the Wnt3a-activation of STF by Ror2 alone and reduced the response mediated by Fzd2 alone or Fzd2 and Ror2 combination by 70% and 80%, respectively. To block the function of Lrp5/6 more broadly, we performed transfection assays with an Lrp5/6 specific inhibitor, Dkk1, and its cofactor, Kremen1 (Krm1) [24]. As shown in Figure 11, Dkk1 and Krm1 almost entirely inhibited STF activity in presence of Ror2 or Fzd2. Similar levels (folds) of inhibition by Dkk1 and Krm1 were observed for Fzd2-Ror2-mediated STF stimulation. Activation of the canonical Wnt signaling through Fzd-Lrp5/6 can also be inhibited intracellularly by GSK3beta. GSK3beta binds to APC and Axin to facilitate beta-catenin phosphorylation and subsequent degradation. When a Xenopus GSK-3, (Xgsk-3), expression plasmid was used in the cotransfection studies, Wnt3a-stimulated STF response decreased significantly in H441 cells transfected with either Ror2 alone, Fzd2 alone or Fzd2 plus Ror2 (Figure 12). Thus, the sum of these results is consistent with a model in which Ror2 potentiates Fzd2-Lrp5/6 in mediating Wnt3a stimulation of the canonical Wnt signaling.

## Discussion

In this study, we report that Ror2 positively modulates the canonical Wnt signaling in mammalian cells. This function occurs through cooperation with Fzd2 and requires the CRD domain and the cytoplasmic domain of Ror2. However, unlike other activities of Ror2, its interaction with Fzd2 in mediating canonical Wnt signaling is independent of its PRD domain. Furthermore, the cooperative effect of Ror2 and Fzd2 is subject to inhibition by Dkk1 and Krm1, which indicates that Ror2 potentiates Fzd2 and Lrp5/6-mediated activation of the canonical Wnt signaling pathway.

Wnt signaling involves multiple mediators within a macromolecular complex and multilayered network of interactions. At the receptor level, 10 Fzds, two Lrps and members of the receptor tyrosine kinase (RTK) family mediate the signal from 19 Wnt ligands [4,25]. To understand the mechanism of Wnt signaling, much of the studies have utilized cell lines that readily respond to Wnt ligands and express high levels of endogenous Fzds. However, it is likely that high endogenous Fzds may complicate the interpretation of experimental results and mask the low affinity interactions at the receptor level. To avoid interference from endogenous Fzds, studies have employed *Drosophila *S2 cells, which exhibit low level Fzd expression [22,26]. Since Wnt signaling in mammalian cells is comparatively more complicated, a mammalian cell line with low Fzd background would provide a useful model for deciphering the complex mechanisms of signaling at the receptor level. We show for the first time that the human lung carcinoma H441 cell line represents a unique model with low-level endogenous Fzd expression. In addition, transient expression of Fzd cDNA alone is sufficient to establish robust Wnt pathway responsiveness to Wnt3a as assayed by STF activation. Thus, H441 cells provide a unique and useful cell model to investigate Wnt signaling.

The role of Ror2 in Wnt5a-mediated activation of the non-canonical pathways has been well established. These include activation of JNK [9], inhibition of Wnt3a-stimulated canonical pathway [4], as well as mediation of Wnt5a-induced cell migration [23]. The current study revealed the role of Ror2 in the canonical pathway. Using H441 cells, the results of our studies clearly demonstrate that Ror2 specifically cooperates with Fzd2, but not Fzd7 to mediate Wnt3a stimulation of the canonical pathway. Physical interactions between Fzd2 and the N-terminal extracellular CRD domain of Ror2 were recently reported [9]. This domain also interacts with Wnt5a [9] and is required for Ror2-mediated cell migration [23]. The proline-rich, PRD domain of Ror2 associates with casein kinase 1ε (CK1ε) which catalyzes phosphorylation of Ror2 at Ser/Thr rich domain, (S/T). This in turn leads to Ror2 auto-phosphorylation at tyrosine residues in PRD [6]. The PRD domain is important for phosphorylating G protein-coupled receptor kinase 2 [6] as well as Ror2-mediated cell migration [23]. The C-terminal intracellular region of Ror2 is also required for enhancing Wnt5a mediated inhibition of canonical Wnt signaling [4]. In the current study we found that the PRD and the S/T domains are not necessary for cooperative function with Fzd2 in mediating canonical Wnt signaling. An Ror2 mutant construct that lacks the PRD and S/T domains functions even more efficiently in its cooperation with Fzd2. This indicates that although PRD and S/T domains are critical for non-canonical Wnt signaling in mammalian cells [4,6,9,23], they are not required for activating canonical Wnt signaling through cooperation with Fzd2.

The sum of the observations outlined above demonstrates Ror2 as a positive modulator of the canonical Wnt signaling at least within a particular context. In this light, Ror2 can be deemed as a key "dual" or multi-functional membrane protein along the Wnt signal transduction pathway in mammalian cells. This multi-functionality is not uncommon for factors participating in Wnt signaling. For example, Wnt5a can activate or inhibit the canonical Wnt signaling depending on the presence of Fzd4 or Ror2, respectively [4]. Members of the Dishevelled family (Dvl) can mediate either canonical or non-canonical Wnt pathways. The C-terminal DEP domain of Dvl is essential for activating PCP pathway, while both N-terminal DIX domain and the C-terminal DEP domain of Dvl are required for activating canonical Wnt signaling [27,28]. A similar functional feature was also found in Ryk, a new member of the Wnt signaling pathway. During mammalian central nervous system development Ryk-Wnt (Wnt1 and Wnt5a) interactions mediate chemorepulsive axon guidance, possibly through the beta-catenin independent PCP pathway [25,29]. Ryk can also mediate the Wnt3a stimulated activation of beta-catenin/Tcf pathway in 293T cells [30].

Previous studies have established that the coupling of Lrp and Fzds through their extracellular domains with Wnt ligands stimulates the canonical or beta-catenin pathway [5,31]. Upon activation of the Wnt pathway, the intracellular domain of Lrp6 is phosphorylated by GSK3 and casein kinase 1, which provides a docking site for Axin to the cell membrane [32,33]. Removing Axin from the cytoplasmic complex of APC/Axin/GSK3 leads to stabilization of beta-catenin. As one of the potential mechanisms of Ror2 in modulating canonical Wnt signaling, Ror2 may interact with Fzd2 which couples with Lrp5/6 in presence of Wnt3a. This hypothesis is partially supported by the following observations: First, the Ror2-Fzd cooperative function occurs with Fzd2 but not Fzd7, even though individually they mediate similar levels of Wnt3a stimulation of STF, suggesting that specific Fzds (Fzd2) must be present. Secondly, both the function of Ror2 alone and the cooperative function of Ror2 and Fzd2 are sensitive to Lrp5/6 specific inhibitors, Dkk1 and Krm1. Thirdly, Ror2 physically interacts with Fzd2 through their CRD domain [[9], Additional file 1] and cooperative function of Ror2 and Fzd2 was greatly reduced by deletion of the Ror2 CRD domain. This model requires further verification by showing directly the formation of Ror2/Fzd2/Wnt3a/Lrp complex in cell membrane.

The Ror2 function may be subject to regulation by multiple other factors. For example, when high levels of Wnt5a are present, Ror2 binds to Wnt5a to mediate the non-canonical Wnt signaling or inhibit the beta-catenin pathway [4,9]. Also, since not all Fzds cooperate with Ror2 in activating the canonical Wnt pathway (Figure 5), the positive function of Ror2 may be dependent on which Fzd(s) functions as the major receptor(s) in a given cell line. And this is further dependent on the trafficking of Fzds to cell membrane and the affinity of each Fzd for a given Wnt ligand. Finally, Ror2 function may be subject to the activity of as yet unknown inhibitors. All of the latter may contribute to a potential explanation for the observation that in A549 cells Ror2 failed to further stimulate the canonical Wnt signaling. This is currently under investigation.

The intracellular domain of Ror2 is composed of the tyrosine kinase domain and PRD domain flanked by S/T domains. We have shown that neither the PRD and S/T domains nor the tyrosine kinase activity is necessary for Ror2 to mediate canonical Wnt signaling. However, the intracellular domain of Ror2 appears not to be entirely dispensable, suggesting that presence of the tyrosine kinase domain but not the tyrosine kinase activity is important.

Different combinations of Wnt ligands and mediators can affect different pathways [4]. Whether Ror2 is involved in non-canonical or canonical Wnt signaling in a given cell may depend on the availability and abundance of its cofactors such as Wnt5a, Fzd2, Fzd4. For example, in presence of Fzd2 and Lrp5/6, Ror2 facilitates Wnt3a to stimulate STF (Figures 5 and 10). However, in presence of high levels of Wnt5a, Ror2 facilitates the antagonistic effects of Wnt5a on Wnt3a [4]. The functional complexities of Ror2 and other Wnt related molecules determine the complex mechanism of Wnt signaling and explain the multifaceted function of Wnt signaling in various cellular functions.

## Conclusion

We have demonstrated that Ror2 modulates Wnt3a-activation of canonical Wnt signaling in H441 cells. In this role, Ror2 cooperates with Fzd2 but not Fzd7 and its activity is dependent on Lrp5/6. We have further shown that the CRD and the tyrosine kinase domains, but not the PRD domain and tyrosine kinase activity of Ror2 are required for mediating the Wnt3a-activation of canonical Wnt signaling. These findings demonstrate the multifunctional properties of Ror2 in canonical and non-canonical Wnt signaling and support a functional scheme whereby regulation of Wnt signaling is achieved by cooperative functions of multiple mediators.

## Methods

### cDNA constructs

cDNA constructs for wild-type Ror2 (Ror2WT) and Ror2 mutants were described previously [6,23] and illustrated in Figure 7. All constructs were generated with pcDNA3 as backbone and with a FLAG Tag at 3' end. Relative expression level of these mutants in mammalian cell lines was previously confirmed [23]. Full-length human cDNA of Fzd2 and Fzd7 were purchased from B-Bridge international, Inc (CA) and verified by sequencing. A 2.4-kb EcoRI/XhoI fragment containing the complete coding region of hFzd2 was cloned into EcoRI/XhoI sites of pcDNA3.1 (Invitrogen Life Technologies, Inc) and designated pcDNA3.1-hFzd2. A 3.5-kb EcoRI/XhoI fragment containing the complete coding region of hFzd7 was cloned into EcoRI/XhoI sites of pcDNA3.1 (Invitrogen Life Technologies, Inc) and designated pcDNA3.1-hFzd7. Expression construct for human Fzd6 driven by CMV promoter was purchased from Origene (Rockville, MD). SuperTopFlash, STF, that contains the luciferase coding region driven by minimal TA viral promoter, plus eight LEF/TCF response elements was kindly provided by Dr. Randall Moon (University of Washington). Xgsk-3 expression construct was kindly provided by Dr. Kimelman (University of Washington). cDNA for Dkk1 and Krm1 were kindly provided by Dr. Christof Niehrs (Deutsches Krebsforechungszentrum, Germany).

### Cell culture, transient transfection assay and conditioned media

Human pulmonary carcinoma H441 and A549 cell lines (ATCC) were maintained in RPMI 1640 or F-12K Nutrient media (Invitrogen Life Technologies, Inc), respectively, containing 10% fetal bovine serum and 1% penicillin-streptomycin. All plasmids used in transfection studies were purified on QIAGEN columns (Qiagen). Transient transfection of H441 and A549 cells was performed with SuperFect (Qiagen) as described previously [11]. Twenty-four hours after transfection, cells were treated for 16 hours with Wnt3a- or Wnt5a- conditioned media (Wnt3aCM or Wnt5aCM) at 1:7 or 1:3 dilutions in serum free media as indicated in each figure. Conditioned media for Wnt3a and Wnt5a were prepared from cultures of L Wnt-3A and L Wnt-5A cells (ATCC) following the protocol from the supplier. Activity of Wnt3a-conditioned medium was compared with that of purified recombinant Wnt3a protein purchased from R&D systems [Additional file 2]. The control conditioned medium (L-CM) was prepared under the same condition as Wnt3aCM and Wnt5aCM from the parental cell line (ATCC CRL-2648) cultured simultaneously with L Wnt-3A or L-Wnt5a and used at the same dilutions. Supernatants of the transfection cell extracts were then prepared with a kit from Promega and used for luciferase assay and beta-galactosidase assay as described previously [11]. Luciferase activity was normalized by beta-galactosidase activity for each transfection. Average of normalized relative luciferase activity was shown with the error bar representing standard deviation. Data represent at least three independent experiments. Significance of the difference in data obtained by transfection assays was determined by Student's t test.

Transfection of H441 cells with siRNA was performed with TransMessenger transfection reagent (Qiagen) following manufacture's instructions. Briefly, for each transfection 6-μl of TransMessenger and 4-μl of Enhancer R were mixed with 1.2-μg of siRNAs and 1.2-μg of plasmid DNAs including *pSV-β*-*gal *(Promega, WI), STF, Ror2WT and Fzd2. After incubation at room temperature for 10 minutes, the transfection complexes were added to H441 cells in 12 well plates. After 3 hours, the transfected cells were washed, cultured, and treated as described above and collected for luciferase and beta-galactosidase assays. SiRNAs for Lrp6 were purchased from Ambion, Inc.

### RNA isolation and Reverse transcription polymerase chain reaction (RT-PCR)

Total RNA was isolated from H441 or A549 cells by using TRIzol reagent (Invitrogen Life Technologies, Inc). cDNA was prepared by using the "SuperScript" kit from the same company. PCR was performed in 50 mM KCL, 10 mM Tris, pH 8.3, 1 mM dNTP, 0.5 μM each primer, 2 units Taq DNA polymerase and 1.3 mM MgCl_2_. The PCR conditions were 94°C for 5 min for 1 cycle followed by 28 (for Fzds) or 32 (for Rors and Lrps) cycles of 94°C for 1 min, 55° to 57°C for 1 min, 72°C for 1 min, with a final extension cycle of 72°C for 7 min. Reaction products were resolved by electrophoresis on 2% agarose gels and stained with ethidium bromide for visualization.

### Sequences of PCR primers

Primer sequences and the expected size (in parenthesis) of the products are as follows.

hFzd1 (343 bp): 5'-GTGCCAATCCTGACATCTCGA-3' (forward), 5'-TAGCTCCTTTGCAATACTCCG-3' (backward);

hFzd2 (321 bp): 5'-GAAAAGCTGGAGCGGCTCAT-3' (forward), 5'-TGGTGAGGCGAGTGTAGAACT-3' (backward);

hFzd3 (322 bp): 5'-CCTATTACCTTGAGGATGTGC-3' (forward), 5'-TATGGCTCATCACAATCTGGG-3' (backward);

hFzd4 (425 bp): 5'-ACCAAGGCAGCATCTAGCAG-3' (forward), 5'-ACTACAGTCGGCACTCAATA-3' (backward);

hFzd5 (411 bp): 5'-GACTGTCTGCTCTTCTCGG-3' (forward), 5'-GGCACATGGGCACCGTGAT-3' (backward);

hFzd6 (366 bp): 5'-CTGATGGGTCATTATGACCAG-3' (forward), 5'-TCTTAAGATGCCTTGGACACC-3' (backward);

hFzd7 (383 bp): 5'-TACTGAGAAGTGACCTGGAAG-3' (forward), 5'-TTTGACCACTGCTTGACAAGC-3' (backward);

hFzd8 (325 bp): 5'-TGCAGCGAAGGGACACTTGA-3' (forward), 5'-AGAGGTTCTCCCAGGTGAAAT-3' (backward);

hFzd9 (333 bp): 5'-AGTACGTGGAGAAGAGCCG-3' (forward), 5'-GTGCAGCCCGTGTTCTCCA-3' (backward);

hFzd10(334 bp): 5'-CAGGATGCTGTGATACACTGA-3' (forward), 5'-TTTCCTCTGCAGGGATGCC-3' (backward);

hRor1 (138 bp): 5'-GGCTGAAACTGCCAAACTGT-3' (forward), 5'-GGTAGTCCACACCTGTGCTGT-3' (backward);

hRor2 (120 bp): 5'-CCTGGACACGACAGACACTG-3' (forward), 5'-AAGTTATGATTTGGGATGTGC-3' (backward);

hLrp5 (121 bp): 5'-CGAATCGAATTGAGGTGTCA-3' (forward), 5'-CACCCCATTCAGTCCAATACA-3' (backward);

hLrp6 (129 bp): 5'-ATCAACGTCCACAGGCTGA-3' (forward), 5'-CCTGCATGTTGGTGAAGTACA-3' (backward);

hKrm1 (494 bp): 5'-GTCCAACAAACTCACCATACAAACT-3' (forward), 5'-

AATACAAGATGACGAAGTCCAGAGA-3' (backward);

### Western blot analysis of the membrane proteins

Membrane proteins were isolated by ReadyPrep Protein Extraction Kit (membrane I) (Bio-Rad laboratories, CA) from H441 cells transfected with Ror2WT or Ror2 mutant derivative constructs. Protein concentration in extracts of hydrophobic phase was determined by RC DC Protein Assay kit (Bio-Rad laboratories, CA). Equal amount of protein was loaded onto SDS-PAGE gel and western blot was performed. Monoclonal anti-FLAG antibody (F1804) was purchased from Sigma (MI). Monoclonal anti-Na+/K+-ATPase α 1 antibody (sc-21712) was purchased from Santa Cruz Biotechnology, Inc (CA).

## Abbreviations

Lrp, low-density lipoprotein receptor related protein; Fzd, Frizzled; STF, SuperTopFlash; L-CM, conditioned medium from L cells; Wnt3aCM, conditioned medium from L Wnt3a cells; Wnt5aCM, conditioned medium from L Wnt5a cells; CRD, cysteine-rich domain; TM, transmembrane domain; Tyr-Kinase, tyrosine-kinase domain; S/T, serine and thronine rich domain; PRD, proline-rich domain.

## Authors' contributions

HC, LH, YX, TS, MFV, JL performed the experiments; MN, YM contributed critical reagents, performed experiments, discussed data and reviewed manuscript; CL, PM designed and performed the experiments, analyzed the data and wrote the manuscript. All authors read and approved the final manuscript.

## Supplementary Material

Additional file 1 Interaction of Ror2 with CRD domains of Fzd2 and Fzd7. 293T cells were transfected with expression constructs for Ror2-HA and Fzd2CRD-Flag or Fzd7CRD-Flag. Protein extracts were immunoprecipitated with anti-Flag antibodies (aFlag). Proteins associated with Protein A-sepharose were than determined by western blot with anti-HA antibody (aHA, for Ror2-HA). Click here for file

Additional file 2 Dose response of STF to Wnt3aCM or recombinant Wnt3a protein in H441 cells. H441 cells were transfected with Fzd2 expression construct. Numbers indicate dilution range of Wnt3a CM (left) or protein concentration (ng/ml) of recombinant Wnt3a protein (right). Click here for file
