# Supplementary figures and images for: Ror2 modulates the canonical Wnt signaling in lung epithelial cells through cooperation with Fzd2
Source: BMC Mol Biol. 2008 Jan 23;9:11. doi: 10.1186/1471-2199-9-11 (PMC2254434; doi:10.1186/1471-2199-9-11)

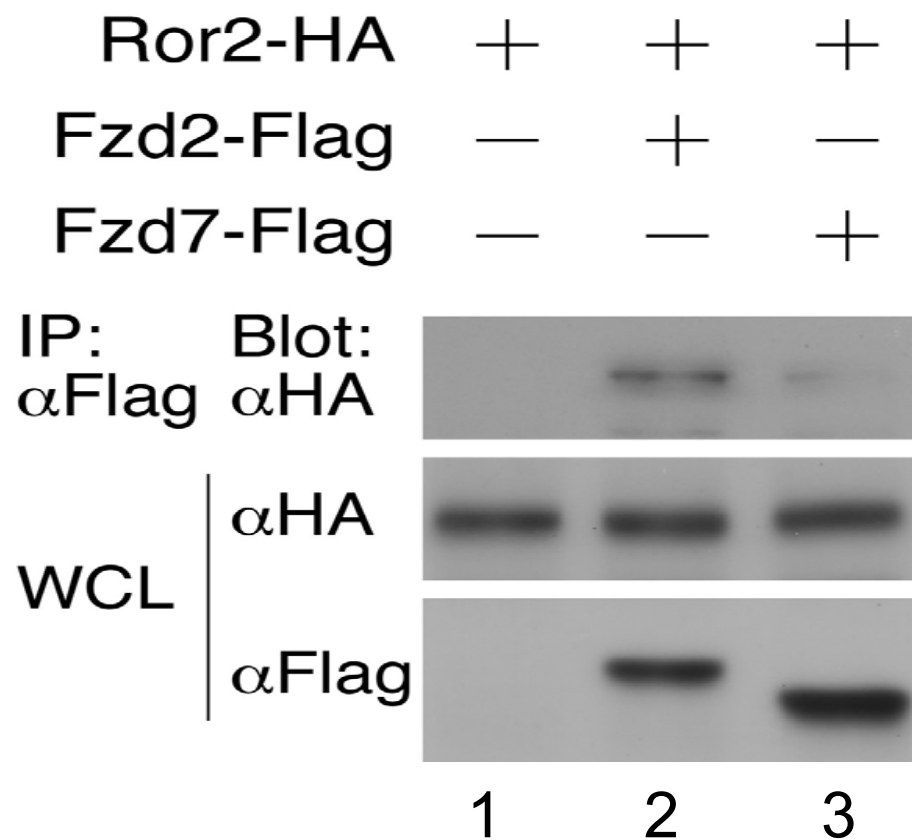

Supplement: Additional file 1 — Interaction of Ror2 with CRD domains of Fzd2 and Fzd7. 293T cells were transfected with expression constructs for Ror2-HA and Fzd2CRD-Flag or Fzd7CRD-Flag. Protein extracts were immunoprecipitated with anti-Flag antibodies (aFlag). Proteins associated with Protein A-sepharose were than determined by western blot with anti-HA antibody (aHA, for Ror2-HA). [file 1471-2199-9-11-S1.pdf]

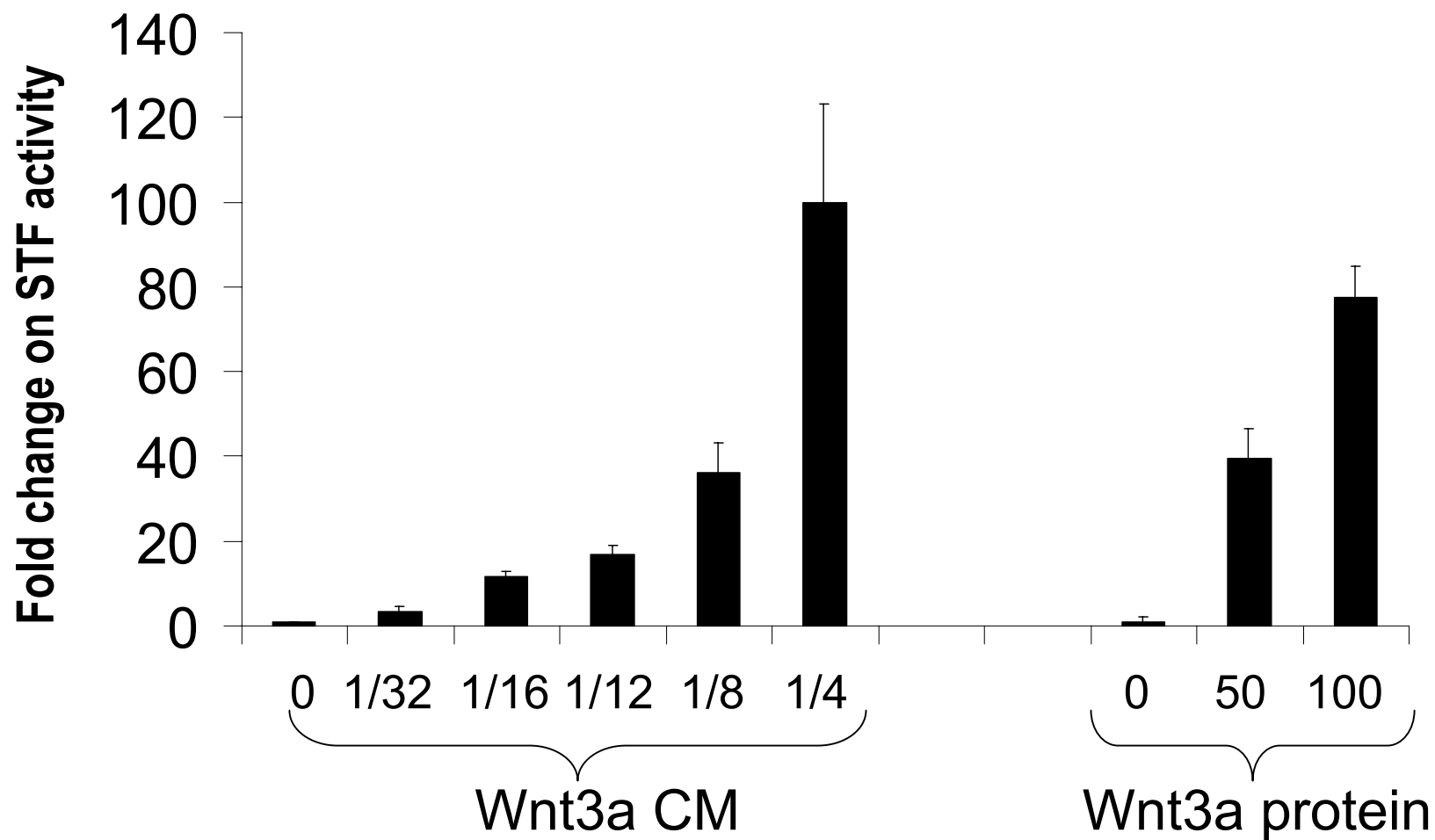

Supplement: Additional file 2 — Dose response of STF to Wnt3aCM or recombinant Wnt3a protein in H441 cells. H441 cells were transfected with Fzd2 expression construct. Numbers indicate dilution range of Wnt3a CM (left) or protein concentration (ng/ml) of recombinant Wnt3a protein (right). [file 1471-2199-9-11-S2.pdf]
